# Improved Growth of *Lactobacillus bulgaricus* and *Streptococcus thermophilus* as well as Increased Antioxidant Activity by Biotransforming Litchi Pericarp Polysaccharide with *Aspergillus awamori*

**DOI:** 10.1155/2013/413793

**Published:** 2013-01-03

**Authors:** Sen Lin, Lingrong Wen, Bao Yang, Guoxiang Jiang, John Shi, Feng Chen, Yueming Jiang

**Affiliations:** ^1^Key Laboratory of Plant Resource Conservation and Sustainable Utilization, Agriculture and Resource Plant Center, South China Botanical Garden, Chinese Academy of Sciences, Guangzhou 510650, China; ^2^College of Life Sciences, University of Chinese Academy of Sciences, Beijing 100039, China; ^3^College of Light Industry and Food Sciences, South China University of Technology, Guangzhou 510640, China; ^4^Guelph Food Research Center, Agriculture and Agri-Food Canada, 93 Stone Road West, Guelph, ON, Canada N1G 5C9; ^5^Department of Food, Nutrition and Packaging Sciences, Clemson University, Clemson, SC 29634, USA

## Abstract

This study was conducted to increase the bioactivity of litchi pericarp polysaccharides (LPPs) biotransformed by *Aspergillus awamori*. Compared to the non-*A. awamori*-fermented LPP, the growth effects of *A. awamori*-fermented LPP on *Lactobacillus bulgaricus* and *Streptococcus thermophilus* were four and two times higher after 3 days of fermentation, respectively. Increased 1,1-diphenyl-2-picrylhydrazyl radical scavenging activity and DNA protection activity of litchi pericarp polysaccharides were also achieved after *A. awamori* fermentation. Moreover, the relative content of glucose and arabinose in LPP after fermentation decreased from 58.82% to 22.60% and from 18.82% to 10.09%, respectively, with a concomitant increase in the relative contents of galactose, rhamnose, xylose, and mannose. Furthermore, lower molecular weight polysaccharides were obtained after *A. awamori* fermentation. It can be concluded that *A. awamori* was effective in biotransforming LPP into a bioactive mixture with lower molecular weight polysaccharides and higher antioxidant activity and relative galactose content.

## 1. Introduction

Litchi (*Litchi chinensis *Sonn.) is a tropical-to-subtropical fruit, with a large amount of polysaccharides in its pericarp, pulp, or seed. As a major byproduct of litchi fruit, the pericarp accounts for approximately 16% of the whole fresh fruit and contains a significant amount of polysaccharides. Due to the health benefits, plant polysaccharides have become a desirable supplement in the functional food industry [1]. *In vitro* studies have indicated that polysaccharides possess broad biological activities including antitumor [2], prebiotic activity [3], and antioxidant capability [4], along with immunostimulating and anti-inflammatory properties [5]. Furthermore, the polysaccharide from *Lentinus edodes*, *Tremella fuciformis*, and *Astragalus membranaceus* could selectively enrich beneficial bacterial species such as *lactobacilli* and *bifidobacteria* in an *in vivo* study [6]. These biological activities of polysaccharides are closely related to their structural characteristics, such as molecular weight and monosaccharide composition. Chen et al. [7] reported the structures of two polysaccharides (LSP I and LSP II) from litchi seeds, with monosaccharide composition of LSP I being mainly glucose (57.3%), galactose (29.7%), and mannose (6.5%) while LSP II consisting of glucose (19.5%), galactose (24.3%), fructose (38.2%), and mannose (10.7%). Kong et al. [8] investigated the polysaccharides present in litchi pulp and determined a polysaccharide composition consisting primarily of glucose, galactose, and arabinose. Yang et al. [9] identified structural information on a polysaccharide from the litchi pericarp. The polysaccharide exhibited a molecular weight of 14 kDa with 65.6% mannose, 33.0% galactose and 1.4% arabinose, and 8.7% of (1-2)-glycosidic linkage, 83.3% of (1–3)-glycosidic linkage, and 8.0% of (1–6)-glycosidic linkage. The polysaccharide also exhibited high superoxide radical scavenging activity.

Previous *in vitro* studies indicated that low molecular weight polysaccharides or hydrolyzed oligosaccharides can improve prebiotic effect [10–12] and increase antioxidant activity [4, 8], but more studies are needed to better understand the structure/activity relationship of these polysaccharides. Currently, biotransformation of plant byproducts by microorganisms or enzymatic engineering is an effective means of producing more bioactive compounds as it can avoid deficiency in chemical modification. Increased biological activity of polysaccharides from agricultural byproducts by enzyme treatment has been reported [13]. However, due to the substrate selectivity of enzyme, the heterogeneity in monosaccharide composition and the structural diversity of plant polysaccharides and bioconversion efficiency by purified enzyme have been confirmed to be low. The combined treatment of several enzymes can partly resolve this problem [13] but it is difficult to choose the appropriate enzymes when the polysaccharide is not clearly characterized. Therefore, an attention to the efficient bioconversion of polysaccharides by microorganisms will be paid as the microorganism contains a multifunctional enzyme system. *Aspergillus awamori* is a filamentous fungus belonging to the *Aspergillus* genus and contains an abundance of hydrolytic enzymes including xylanase, pectinase, and *β*-glucosidase [14]. Thus, application of *A. awamori* may be an effective enzyme source for production of low molecular weight polysaccharides. The objective of this present study was to investigate the structural characteristic related to antioxidant activity and the growth effect of litchi pericarp polysaccharides (LPPs) on *Lactobacillus bulgaricus* and *Streptococcus thermophilus* after *A. awamori* fermentation.

## 2. Materials and Methods

### 2.1. Litchi Pericarp and Microorganism

Fresh fruit of litchi (*Litchi chinensis *Sonn.) cv. Huaizhi were harvested from a commercial orchard in Guangzhou, China. The fruit were washed with distilled water and then peeled manually. The pericarp was collected, then dried outdoors, and finally ground into powder.

The microorganism *Aspergillus awamori* GIM 3.4 was obtained from Guangdong Culture Collection Center, Guangzhou, China. The fungus was cultured for 3 days on potato dextrose agar (Guangdong Huankai Microbial Science & Technology Co.) at 30°C. *A. awamori* spores were collected by washing the agar surface with sterile distilled water containing 0.1% Tween 80. The spore suspension was adjusted to a concentration of ca. 10^6^ cfu/mL using sterile distilled water and then used to biotransform LPP.

### 2.2. Chemicals

All chemicals to prepare the Czapek-Dox medium were obtained from Guangzhou Reagent Co. 1,1-Diphenyl-2-picrylhydrazyl (DPPH), xylose (Xyl), arabinose (Ara), glucose (Glc), galactose (Gal), fructose (Fru), mannose (Man), fructose (Fru), and rhamnose (Rha), and dextrans standards were purchased from Sigma Chemical Company (St. Louis, MO, USA).

### 2.3. Extraction of Polysaccharides from Litchi Pericarp

Polysaccharides were extracted from litchi pericarp according to the method of Kong et al. [8] with minor modifications. Briefly, litchi pericarp (150 g) was immersed into distilled water (1000 mL) after washing with 80% alcohol twice to remove monosaccharides and oligosaccharides. The extraction was repeated three times at 80°C for 4 h, and each extraction solution was filtrated through Whatman No. 1 paper. The filtrates were collected, combined, and then concentrated to 100 mL under vacuum. The proteins in the extract solution were removed using the sevag reagent, and the polysaccharides were then precipitated with four volumes of ethanol overnight at 4°C. The precipitate was collected by centrifugation at 10,000 g for 20 min, then washed successively with ethanol and ether, and finally dried under vacuum at 65°C to obtain the crude polysaccharides.

### 2.4. Fermentation

For the fermentation, polyurethane foam (3 g) was placed into Erlenmeyer flasks (250 mL) and then mixed with the modified Czapek-Dox medium (10 mL) containing NaNO_3_ (30 mg), K_2_HPO_4_ (10 mg), KCl (5 mg), MgSO_4_·7H_2_O (5 mg), FeSO_4_ (0.1 mg), sucrose (0.3 g), LPP (0.3 g), and distilled water (10 mL). The medium was sterilized for 30 min at 121°C and then incubated with l mL of fresh *A. awamori* spore suspension at 28°C. The fermented products were collected by adding 50 mL of distilled water and holding the mixture fro 4 h at 70°C prior to filtering. This extraction was repeated, and the filtrates were collected, combined, and then concentrated under vacuum at 65°C. A control sample was prepared by incubating the fresh spore suspension (1 mL) with the previous culture medium but without LPP.

### 2.5. Molecular Weight Determination of LPP

The molecular weight (MW) of LPP was determined by gel permeation chromatography (GPC) by the method of Yang et al. [15]. The analysis was performed using high-performance liquid chromatography (Waters 5215, Milford, MA) equipped with a TSKG-5000 PW xL gel column (7.8 × 300 mm) and a TSK G-3000 PW xL gel column (7.8 × 300 mm) coupling with a model 2414 refractive index detector and a Breeze GPC workstation. Samples were eluted with 20 mM KH_2_PO_4_ (pH 6.0) at a flow rate of 0.6 mL/min. The column temperature was maintained at 35°C, and the injection volume was 30 *μ*L. Dextrans with different molecular weights (4400, 9900, 21,400, 43,500, 124,000, 196,000, 277,000, and 845,000 Da) were employed as standards. The molecular weights of the fermented and nonfermented LPPs were obtained from the equation based on the elution volume of standard dextrans to their log molecular weights.

### 2.6. Analysis of Monosaccharide Compositions of LPP

A gas chromatograph (GC-2010, Shimadzu, Shanghai, China) equipped with a RTX-5 capillary column and a flame ionization detector was employed for the identification of the monosaccharides in the fermented and nonfermented LPPs. The fermented and nonfermented LPPs (10 mg) were hydrolyzed for 6 h with 10 mL of 2 M trifluoroacetic acid (TFA) at 120°C [16]. After hydrolysis, LPP was dried at 65°C using a rotary evaporator (RE52AA, Yarong Instrument Co., Shanghai, China). The released monosaccharides were derivatized with trimethylsilyl reagent according to the method of Guentas et al. [17]. Briefly, the hydrolyzed products were mixed for 5 min with 2 mL pyridine, 0.4 mL hexamethyldisilazane, and 0.2 mL trimethylchlorosilane at 25°C. After centrifugation at 13,200 g for 15 min, the trimethylsilyl derivative (1 *μ*L) was analyzed by gas chromatography. The temperature program started at 130°C, held for 1 min, increased to 180°C at 2°C/min, held for 3 min, increased to 220°C at 10°C/min, and held for 3 min [18]. The gas chromatograph was run in the splitless mode. Inositol was used as the internal standard. The monosaccharides were identified and quantitated by comparing retention times and peak areas of the standards, respectively.

### 2.7. Measurement of DPPH Radical Scavenging Activity

The method described by Sánchez-Moreno et al. was used to assess the DPPH radical scavenging activity of the fermented and nonfermented LPPs [19]. Briefly, an aliquot (0.1 mL) of these samples at 0.05, 0.1, 0.2, 0.5, and 1 mg/mL was mixed with 2.9 mL of 0.1 mM DPPH in methanol. The absorbance was measured at 517 nm after 30 min at 25°C. The control was carried out with water instead of the tested sample while methanol instead of the DPPH solution was used for the blank. The DPPH radical scavenging activity (%) of the tested sample was calculated as [1 − (absorbance of sample − absorbance of blank)/absorbance of control)] × 100.

### 2.8. Protection Effect against DNA Breakage

The protective abilities of the fermented and nonfermented LPP on supercoiled DNA damage were investigated by the method of Kang et al. [20]. *Escherichia coli* DH5a cells were transformed with pUC19 plasmid DNA and then grown overnight in the Luria-Bertani (LB) medium containing ampicillin (50 *μ*g/mL) at 37°C. Plasmid DNA was purified using the UNIQ-10 Plasmid Kit (Wuhan Sikete Science & Technology Development Co. Ltd., Wuhan, China). Evaluation of the protection activity against Fenton-reaction-mediated DNA breakage was conducted using supercoiled plasmid DNA [21]. Five microliters of 100 mM Tris-HCl buffer (pH 7.5), 2 *μ*L of 50 mM hydrogen peroxide, 10 *μ*L of 0.2 *μ*g/*μ*L plasmid DNA, and 1 *μ*L of samples at 0.05, 0.1, 0.2, 0.5, and 1 mg/mL were mixed. The reaction was initiated by adding 2 *μ*L of 5 mM ferrous sulfate into the mixture. After 15 min at 30°C, the reaction was stopped by adding 10 *μ*L of stop solution containing 8 M urea, 50% sucrose, 50 mM EDTA, and 0.1% bromophenol blue and then separated by 1% agarose gel electrophoresis for 40 min under 120 V condition. The agarose gel was stained with 0.05% (w/v) ethidium bromide and then analyzed with an image analyzer (Image station 2000 R, Kodak, New York, USA).

### 2.9. Growth Effects of LPP on *Lactobacillus bulgaricus* and *Streptococcus thermophilus*

The growth effects of the fermented and nonfermented LPPs on *L. bulgaricus* and *S. thermophilus* were conducted by the method of Yang et al. [3]. An aliquot (100 *μ*L) of *L. bulgaricus* or *S. thermophilus* cells in 10% (v/v) glycerol/water solution was pipetted to Man, Rogosa, and Sharp (MRS) agar plate containing 10 g/L protease peptone, 10 g/L beef extract, 5 g/L yeast extract, 1 g/L Tween 80, 2 g/L ammonium citrate, 5 g/L CH_3_COONa, 0.1 g/L MnSO_4_, 0.05 g/L MgSO_4_, 2 g/L K_2_HPO_4_, 20 g/L glucose, and 13 g/L agar, then inoculated at 37°C, and finally incubated for 48 h. A single colony was then transferred to a tube containing 5 mL of sterilized MRS broth (121°C, 20 min) and then cultured for 24 h at 37°C by shaking at 130 rpm. To comparatively evaluate the growth effect, an aliquot (100 *μ*L) of *L. bulgaricus* or *S. thermophilus* cell suspension (starting concentration of 1 × 10^6^ cfu/mL) was mixed with 2.5 mL of MRS broth and LPP samples at 0, 50, 100, and 200 *μ*g/mL. The mixture was incubated for 24 h at 37°C with shaking at 130 rpm. After dilution, the mixture (100 *μ*L) was added to MRS agar plate and then cultured at 37°C for 48 h. The final cell concentration was estimated by counting the colonies on the plate.

### 2.10. Statistical Analysis

Data were expressed as mean ± standard deviations (SD) and then analyzed by OriginPro 8 (OriginLab Corporation, Massachusetts, USA). Graphs were made using Sigmaplot (Systat Software Inc., San Joes. CA). One-way analysis of variance (ANOVA) and the Tukey's multiple comparisons were carried out to test for significant differences between the means. Differences between the means at the 5% level were considered significant.

## 3. Result and Discussion

### 3.1. Effect of *A. awamori* Fermentation on Molecular Weight of LPP

**Figure 1:**
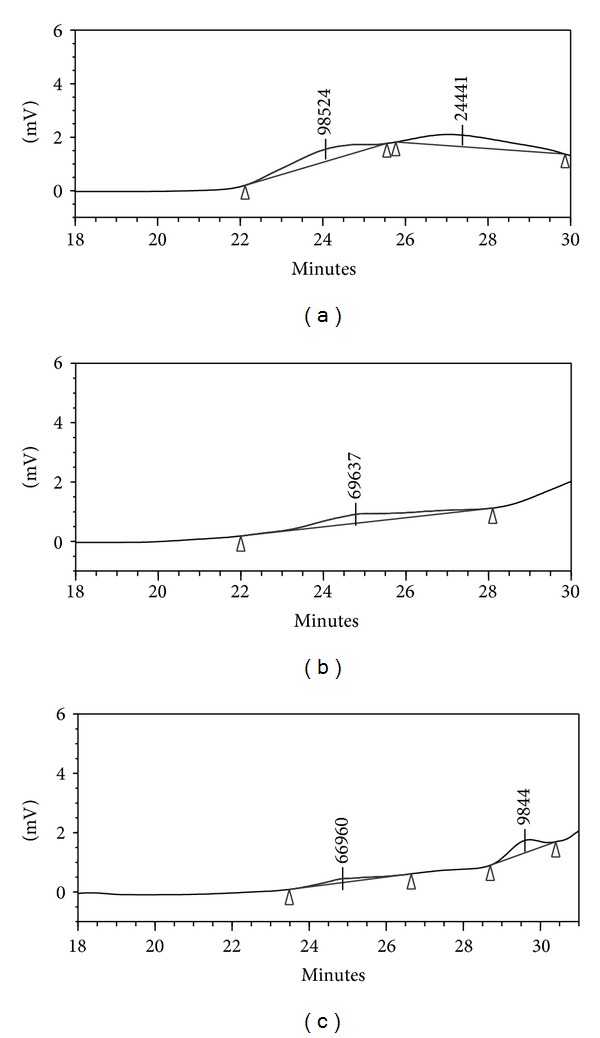
Gel permeation chromatography (GPC) of non-*A. awamori*-fermented LPP (a) and *A. awamori*-fermented LPP after 3 (b) or 6 days (c).

The molecular weight of LPP after fermentation was examined by high-performance gel permeation chromatography, with the equation fitting the standard curve of log MW = 49.5 − 7.75 *V* + 0.459 *V* ^2^ − 0.00938 *V* ^3^ and the correlation coefficient of 0.997 (where MW and *V* represented the molecular weight and the elution volume, resp.). As shown in Figure 1(a), the non-*A. awamori*-fermented LPPs displayed two peaks in the chromatogram, and their molecular weights were estimated to be 98.524 and 24.441 kDa, respectively. After 3 days of fermentation, one band with an estimated MW of 69.736 kDa was observed (Figure 1(b)), which indicated that LPP was degraded. The LPP was further degraded after 6 days of fermentation, and two polysaccharides with lower MW of 66.96 and 9.844 kDa were obtained (Figure 1(c)). The presence of hydrolytic enzymes such as arabinofuranosidase, xylanase, and glucoamylase [22] produced by *A. awamori* may account for LPP degradation.

### 3.2. Effect of *A. awamori* Fermentation on Monosaccharide Compositions of LPP

The monosaccharide profiles of non-*A. awamori*-fermented and *A. awamori*-fermented LPPs after 3 and 6 days of fermentation are shown in Table 1. The non-*A. awamori*-fermented LPP consisted mainly of glucose (58.82%), arabinose (18.78%), galactose (9.99%), rhamnose (5.03%), mannose (3.79%), and xylose (2.49%). The result was different from the previous report by Yang et al. [9], who identified mannose (65.6%), galactose (33%), and arabinose (1.4%) as the major monosaccharides of polysaccharides from litchi seed. In the present, the polysaccharide was from litchi pericarp, and it was precipitated by 80% alcohol, while in the previous report one polysaccharide fraction was purified sequentially by DEAE Sepharose fast-flow column and Sephadex G-50 gel column, which may explain the different information of polysaccharide structure. After fermentation, the relative molar percentages of glucose and arabinose decreased rapidly while those of galactose, rhamnose, mannose, and xylose increased. *A. awamori* can produce a number of arabinanases [22] and glucoamylase [23] that may specifically release arabinose and glucose from plant polymers and then use the hydrolysates as a carbon source [24], which could account for the decreases in the relative percentages of arabinose and glucose content in the fermented LPP. The decreases in relative arabinose and glucose contents in the fermented LPP could also explain the increase in other monosaccharides.

### 3.3. Effect of *A. awamori* Fermentation on DPPH Radical Scavenging Activity of LPP

**Figure 2:**
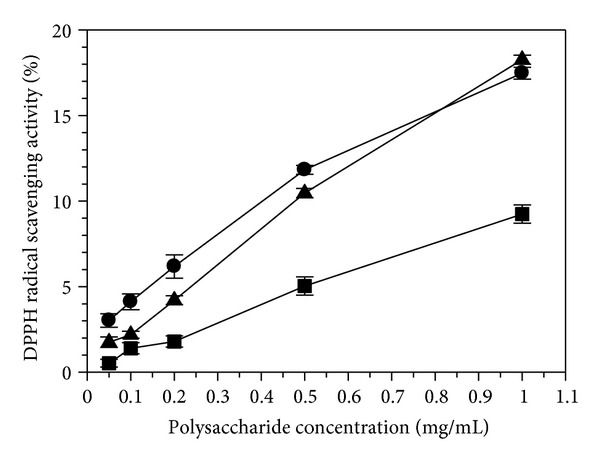
DPPH radical scavenging activity of non-*A. awamori-fermented* LPP (*∎*) and *A. awamori-fermented* LPP after 3 (●) or 6 days (▲). Each value was expressed as the mean ± standard error (*n* = 3).

Epidemiological evidence indicates an inverse correlation between the consumption of plant antioxidants and incidence of several chronic diseases [25]. The DPPH radical scavenging activity of LPP is presented in Figure 2. A dose-dependent effect existed in all three LPP samples. However, the DPPH radical scavenging activity of LPP was relatively low in the present study. For the non-*A. awamori*-fermented LPP, less than 10% of the DPPH radical scavenging activity was observed when a concentration of 1 mg/mL was used, which was consistent with the previous reports in the polysaccharides extracted from litchi seed and soy sauce lees, respectively [3, 6]. After *A. awamori*-fermentation, the LPP exhibited a marked increase in the DPPH radical scavenging activity. After 3 and 6 days of fermentation, the *A. awamori*-fermented LPP had a stronger DPPH radical scavenging activity than the non-*A. awamori*-fermented LPP. When 1 mg/mL was employed, the DPPH radical scavenging activity of the *A. awamori*-fermented LPP increased from 9.24 ± 0.53% on the initial day to 17.47 ± 0.35% on the third day and to 18.27 ± 0.27% on the sixth day, which suggested that the polysaccharide modification influenced greatly the DPPH radical scavenging activity. Previous report of Kong et al. [8] indicated that three fractions of polysaccharides (LFP1, LFP2, and LFP3) from litchi pulp with decreasing molecular weight (LFP1 > LFP2 > LFP3) exhibited an increasing trend in antioxidant activity (LFP1 < LFP2 < LFP3). In the study, Gel permeation chromatography analysis further revealed that lower MW polysaccharides from litchi pericarp can be produced by fermentation, which can account for the increase in the DPPH radical scavenging activity after fermentation. Thus, the increased DPPH radical scavenging activity of the polysaccharides from litchi pericarp can be achieved by biomodification, which will be beneficial to the functional food industry.

### 3.4. Effect of *A. awamori* on DNA Protection Effect of LPP

**Figure 3:**
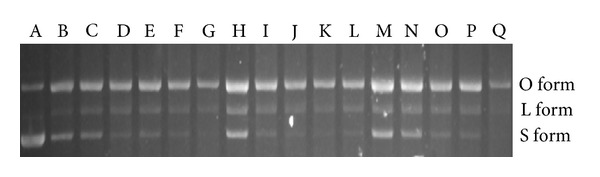
Electrophoretic patterns of plasmid DNA of pUC19 in the presence of non-*A. awamori*-fermented LPP and *A. awamori*-fermented LPPs after 3 or 6 days. A, pUC19; B, C, D, E, F, pUC19, damage solution, and non-*A. awamori*-fermented LPPs at 1, 0.5, 0.2, 0.1, and 0.05 mg/mL; G, pUC19 and damage solution (blank); H, I, J, K, and L, pUC19, damage solution and *A. awamori*-fermented LPPs after 3 days at 1, 0.5, 0.2, 0.1, and 0.05 mg/mL; M, N, O, P, and Q, pUC19, damage solution and *A. awamori*-fermented LPP after 6 days at 1, 0.5, 0.2, 0.1, and 0.05 mg/mL. The damage solution containing 50 mM ^•^OH generated by mixing 2 *μ*L of 50 mM hydrogen peroxide and 2 *μ*L of 5 mM ferrous sulfate. Supercircular DNA was indicated as S form, linear DNA is indicated as L form, and open circular DNA is indicated as O form.

In this study, as litchi pericarp polysaccharide exhibits a relatively low antioxidant activity using the DPPH method (Figure 2), it is needed to use DNA protection activity to assess generally antioxidant activity of litchi pericarp polysaccharides modified by microorganism. Plasmid DNA has three forms that can be separated by agarose gel electrophoresis. These forms are the supercoiled circular form (S form), the open circular form (O form), and the linear form (L form), as shown in Figure 2 (Line A). The S form of DNA is damaged, resulting in from the O form to L form when exposed to reactive oxygen species. Some plant extracts were reported to exhibit the ability to protect DNA from the Fenton-reaction-mediated breakage [20, 26, 27]. In the study, the *A. awamori*-fermented and non-*A. awamori*-fermented LPPs were used to further investigate the DNA protection activity. As shown in Figure 3, the S form of DNA was degraded to produce the O form and L form in the absence of LPP (Line G). It was further degraded into smaller DNA fraction [28] as the band intensity present in Line G was much lighter than Line A (pUC18 without treatment with reactive oxygen species). Compared to Line G, the band of the S form of DNA present in Lines B, H, and M was much brighter. The brightness decreased with decreasing LPP concentration. Thus, the non-*A. awamori*-fermented LPP (Line B to F) and *A. awamori*-fermented LPP after 3 days (Line H to L) and 6 days (Line M to Q) exhibited an increased DNA protection activity against supercoiled breakage after fermentation, which further confirmed that the fermentation enhanced the antioxidant activity of LPP (Figure 2). Overall, the *A. awamori*-fermented LPP possessed higher DNA protection activity. A number of published papers have reported the enhanced DNA protection activity of the polysaccharides from macro fungi [29, 30] and microorganisms [31, 32].

### 3.5. Effect of *A. awamori* Fermentation on Growth Effects of LPP on *L. bulgaricus* and *S. thermophilus*

**Figure 4:**
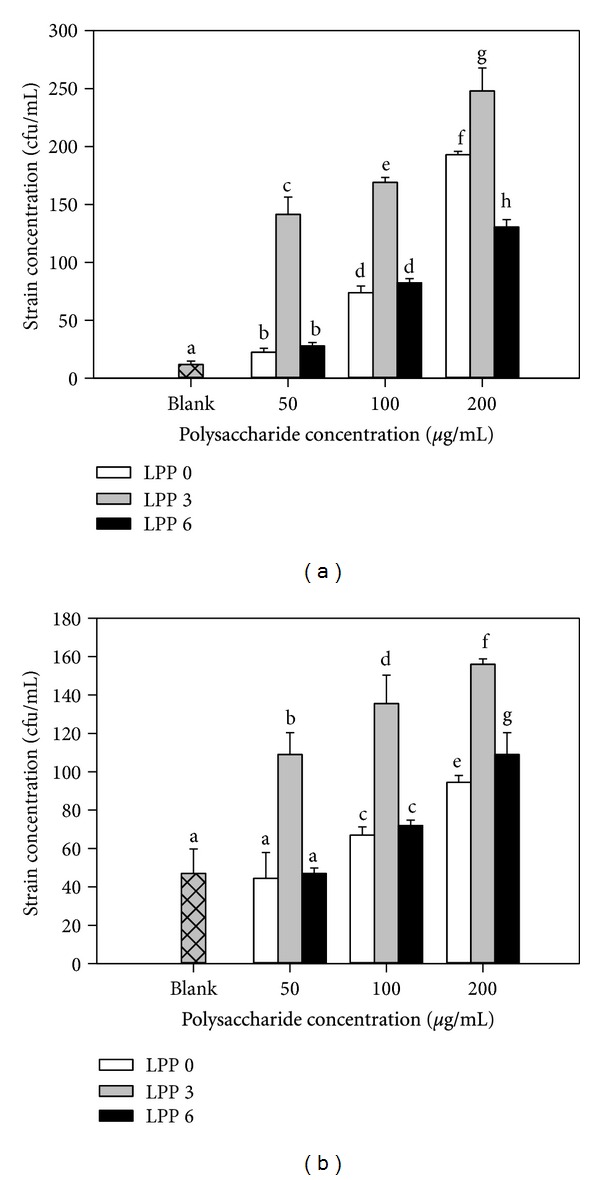
Growth effects of non-*A. awamori*-fermented (LPP 0) and *A. awamori*-fermented LPP after 3 (LPP 3) and 6 days (LPP 6) on *Lactobacillus bulgaricus* (a) and *Streptococcus thermophilus* (b). The blank was culture solution without LPP. Each value was expressed as the mean ± standard error (*n* = 3).

The growth effect of non-*A. awamori*-fermented and *A. awamori*-fermented LPPs on *L. bulgaricus* is presented in Figure 4(a). LPP more significantly promoted (*P* ≤ 0.05) the growth of *L. bulgaricus*. A dose-dependent response was observed in the present study. Compared to the non-*A. awamori*-fermented LPP, the fermented LPP at 50 *μ*g/mL after 3 days of fermentation showed four times higher growth effect, but no significant difference was observed between the non-*A. awamori*-fermented and *A. awamori*-fermented LPPs at between 50 and 100 *μ*g/mL after 6 days of fermentation. Similarly, the *A. awamori*-fermented LPP after 3 days of fermentation showed much higher growth effects on *S. thermophilus* compared with the non-*A. awamori*-fermented LPP (Figure 4(b)). However, the growth effect of the *A. awamori*-fermented LPP after 6 days of fermentation decreased. The present study demonstrated that the *A. awamori*-fermented LPP after 3 days of fermentation with an MW of 69.736 kDa possessed the higher growth effects on *L. bulgaricus* and *S. thermophilus* compared to the non-*A. awamori*-fermented LPP (about 98.524 kDa) and the fermented LPP (about 9.844 Da) after 6 days of fermentation. Previous studies have also indicated that low weight polysaccharides or hydrolyzed oligosaccharides can enhance these effects [10–12].

Large amount of microorganisms including *Bifidobacteria*, *Lactobacilli,* and *Streptococcus* densely populates in human gut. The microorganisms belonging to *Lactobacilli* genus are believed to be beneficial to human health through improving absorption of nutrients, preventing colonisation of pathogens and stimulating immune response in humans [33]. Thus, we evaluated the growth effect of the *Aspergillus awamori*-fermented litchi pericarp polysaccharides on *L. bulgaricus* and *S. thermophilus* in an effort to understand the beneficial ability of the biomodified litchi polysaccharides. However, the relationship between biomodified litchi pericarp polysaccharide and growth effects on *L. bulgaricus* and *S. thermophilus* needs to be further investigated.

## 4. Conclusions

The fermentation of LPP by *A. awamori* can degrade markedly polysaccharide with reduced molecular weight and increased contents of galactose, rhamnose, mannose, and xylose. Increases in growth effect of *L. bulgaricus* and *S. thermophilus*, DNA protection activity, and DPPH radical scavenging activity were obtained after fermentation of LPP by *A. awamori*. This study provided evidence that litchi paricarp polysaccharide can be enzymatically modified to increase its bioactivity. Thus, *A. awamori* fermentation of LPP can be served potentially as a means of increasing the utilization of this readily accessible waste material.

## Figures and Tables

**Table 1:** The relative changes in molar percentages of monosaccharide in non-*A. awamori*-fermented LPP (LPP 0) and *A.  awamori*-fermented LPP after 3 (LPP 3) or 6 days (LPP 6).

Monosaccharide	LPP 0	LPP 3	LPP 6
Ara	18.78 ± 0.46^a^	15.17 ± 1.94^b^	10.09 ± 0.13^c^
Rha	5.03 ± 0.41^a^	6.16 ± 0.92^a^	10.41 ± 0.82^b^
Xyl	2.49 ± 0.38^a^	7.44 ± 0.60^b^	8.42 ± 1.57^b^
Fru	1.47 ± 0.20^a^	6.10 ± 1.57^b^	6.90 ± 0.92^b^
Man	3.79 ± 0.13^a^	6.02 ± 1.17^b^	8.03 ± 1.9^b^
Glc	58.82 ± 1.38^a^	40.89 ± 1.46^b^	22.60 ± 0.98^c^
Gal	9.99 ± 0.08^a^	18.18 ± 0.17^b^	33.98 ± 1.20^c^

Data with different letters within the same row were significantly different (*P* ≤ 0.05).
